# RRM2 expression in different molecular subtypes of breast cancer and its prognostic significance

**DOI:** 10.1186/s13000-021-01174-4

**Published:** 2022-01-05

**Authors:** Manar Ahmed Abdel-Rahman, Mena Mahfouz, Hany Onsy Habashy

**Affiliations:** Department of Pathology, Faculty of Medicine, Mansoura University, El-Gomhoria St., Mansoura, Dakahlia Egypt

**Keywords:** Breast cancer, RRM2, Expression, Prognosis

## Abstract

**Background:**

Breast cancer is one of the most common types of cancer. Ribonucleotide reductase (RNR) is a heterodimeric tetramer consisting of two Ribonucleoside-diphosphate reductase large subunits (RRM1) and two Ribonucleoside-diphosphate reductase small subunits (RRM2). RRM2 is the building subunit of RNR that is important for synthesis of Deoxynucleoside triphosphate (dNTP) during S phase of cell cycle during DNA replication. RRM2 is associated with poor prognosis in lung and colorectal cancer. In breast cancer, increased RRM2 protein level is strongly correlated with large tumour size, positive lymph node and relapse. In this study, we aimed to study expression of RRM2 in breast cancer and to correlate it with different clinicopathological parameters in Egyptian women.

**Material and methods:**

This study was performed by investigating RRM2 protein expression in breast cancer and correlating the results with other clinicopathological variables using immunohistochemistry and tissue microarrays.

**Results:**

About 77% of cases were RRM2 positive. High Ki67 was observed in cases with high RRM2 score. The majority of non-luminal cases expressed RRM2, however this was statistically insignificant. In ER positive group, RRM2 expression was associated with shorter disease free survival with borderline significance.

**Conclusion:**

RRM2 protein expression can help in evaluating outcome of breast cancer patients and could be a potential therapeutic target.

## Introduction

Breast cancer is one of the common and deadly types of cancer [1]. Molecular classification of breast cancer has emerged as a novel way to subclassify breast cancer into new subtypes of breast cancer including luminal A, luminal B, HER2/neu enriched and basal-like breast cancer [2, 3]. Among these subtypes of breast cancer, the luminal tumours, which are most common types, luminal A has the best prognosis compared to luminal B tumours. Her2 tumours have a higher rate of lymph node spread and recurrence especially in early breast cancer (stage I and stage II). Basal-like cancers (BLBC) including triple negative cancers have a high rate of recurrence and poor prognosis [4]. Study of novel biomarkers to characterize these subtypes is of great importance.

Ribonucleotide reductase (RNR) is a heterodimeric tetramer consisting of two Ribonucleoside-diphosphate reductase large subunits (RRM1) and two Ribonucleoside-diphosphate reductase small subunits (RRM2). RRM2 is the building subunit of RNR that is important for synthesis of Deoxynucleoside triphosphate (dNTP) during S phase of cell cycle during DNA replication. RRM2 produces a stable tyrosine radical which is combined to RRM1 cysteine to start the reduction reaction [5]. Knocking down of RRM2 significantly decreased proliferation during S phase of cell cycle [6, 7] and induced tamoxifen resistance in breast cancer by regulating cell growth and DNA damage via the (AKT)-induced protein kinase B reversal [8]. According to Shah et al. [9], inhibition of RRM2 can reverse tamoxifen resistence in vivo and can reduce in vitro invasive potentials of the tumours.

Other investigators have studied the prognostic significance of RRM2 in breast cancer cases, and it was found that increased RRM2 protein levels strongly correlated with large tumour size, positive lymph nodes and relapse. The expression of RRM2 in breast cancer can confer hormonal therapy resistance and an altered ER status. It is well known that targeting oestrogen receptor is one of the important lines of treatment in breast cancer [1, 10, 11].

Other studies found that silencing of RRM2 weakened breast cancer cell invasion and migration by controlling the PI3K signalling pathway [12]. In vitro studies showed that triple negative breast cancer cell lines had a high expression of RRM2 [11]. RRM2 was found to be a strong prognostic marker in evaluating outcome for patients with ER-negative breast cancer [1].

RRM2 was found to be associated with poor prognosis in lung and colorectal cancer [10, 13, 14]. It promotes the invasion of pancreatic adenocarcinoma cells and it was found to be a determinant of malignant cellular behaviour in a wide range of human cancers [15]. It has been found that increased RRM2 expression is associated with increased expression of markers and genes of proliferation in bladder cancer [16]. In addition, RRM2 expression was significantly associated with Ki67 expression and with shorter survival in ovarian cancer [6].

In this study, we aimed to study RRM2 protein expression in different molecular subtypes of breast cancer and to correlate its expression with different prognostic parameters in breast cancer especially ER-positive subgroup.

## Material and methods

This is a retrospective study conducted on cases of invasive breast carcinoma obtained from histopathology laboratory in Oncology Center, Faculty of Medicine, Mansoura University starting from January 2012 excluding the cases received preoperative neoadjuvant therapy and HER2/neu (+ 2) tumours. All clinicopathological data were revised. Histological subtypes (according to the WHO classification 2019), histological grade, TNM staging and presence of local recurrence or distant metastasis were included. Histological grade of tumours was determined according to Nottingham Modification of the Bloom-Richardson Scoring System [17]. The American Joint Committee on Cancer (AJCC) was used as a reference for staging [18].

*Approval of institutional research board of Faculty of Medicine, Mansoura University, code number R.21.03.1250.R1.R2 was obtained.*

### Tissue microarray construction

H&E tissue sections were the guide to select the regions for tissue sampling. Tissue Microarray (TMA) was assembled manually using three cores from each case [19]. Two hunderd cases were available for assessment.

### Immunohistochemistry

4 μm thickness sections were cut from paraffin-embedded tissue microarray. Then deparaffinization was performed using xylene. After that, we rehydrated them with descending grade of alcohol followed by antigen retrieval in P-T link system from DAKO. Rabbit polyclonal primary antibody against RRM2 (NBP1–31661), obtained from NOVUS Biological, Centennial, Co 80,112 USA was used at a dilution rate of 1:50.

(DAB) was used as a chromogen, after that, hematoxylin was applied for counterstaining. Sections of breast cancer were used as a positive control for RRM2, while lymphoid follicles germinal centers were used as a positive control for Ki67. Cores fall rate was minimal and each case was presented by three cores. In case of falling cores, we considered the core that was remaining on the slides.

### Evaluation of immunohistochemistry

For RRM2, only cytoplasmic staining of tumour cells was considered positive with a cut-off more that 10% of tumour cells. Intensity of RRM2 positivity was scored as no staining (0), weak staining (+ 1), moderate staining (+ 2) and intense staining (+ 3) [1]. Ki67 staining was interpreted as low or high using a 14% cut-off and also as a continuous data [20]. Based on immunohistochemical expression of ER, PR, HER2, EGFR and Ki67, tumours were classified into luminal A (ER+ and / or PR+, HER2-, any EGFR and Ki67 < 14%); luminal B, including luminal B/ HER2 negative (ER+ and /or PR+, HER2-, any EGFR and Ki67 > =14%) and luminal B/ HER2 positive (ER+ and /or PR+, HER2+, any EGFR, any Ki67); HER2 enriched (HER2+ and ER−/ PR-) and triple negative subtypes (ER-, PR-, HER2- and any EGFR).

### Statistical analysis

Data were analyzed using the statistical package of social science (SPSS) program for windows (standard version 24). The normality of data was first tested with one sample Kolmogorov-Simonov test.

Qualitative data were described using number and percent. Association between categorical variables was tested using chi-square test while Fischer exact test and Monte Carlo test were used when expected cell count is less than 5. Continuous variables were presented as mean ± standard deviation (SD). Spearman correlation was used to correlate ordinal data. For all above mentioned statistical tests done, the threshold of significance is fixed at 5% level (*p* value). The results were considered significant when *p* value was < 0.05. The smaller the *p* value obtained the more significant were the results. For Ki67 correlations with RRM2 scores, we used Kruskil Wallis test.

## Results

Clinical and pathological characteristics of the entire cohort are illustrated in (Table 1). The mean age of cases included in this cohort was (58.14 ± 12.11, ST). The majority of cancer breast cases were positive for both ER (69%) and PR (62%). However most cases were HER2 negative (77.5%). Median Ki67 expression was 10. Regarding molecular subtypes of breast cancer, the most common subtypes were luminal A (37.5%) followed by luminal B (20%).

**Table 1:** Clinical and pathological characteristics of patient cohort

Socio-demographic data	The studied group (***n*** = 200)
**Age/ years**	
Mean ± SD	58.14 ± 12.11
< 60 y	109 (54.5%)
≥ 60 y	91 (45.5%)
**ER**	
Positive	138 (69.0%)
Negative	62 (31.0%)
**PR**	
Positive	124 (62.0%)
Negative	76 (38.0%)
**HER2**	
Positive (score 3+)	45 (22.5%)
Negative	155 (77.5%)
**Ki 67** Median (Min-Max)	10 (2–60)
**Molecular Class**	
Luminal A	75 (37.5%)
Luminal B	40 (20.0%)
TN BLBC	39 (19.5%)
Her2 Luminal	17 (8.5%)
Her2 enriched	29 (14.5%)
**EGFR**	
Positive	150 (75.0%)
Negative	50 (25.0%)
**Histological type**	
Ductal	173 (86.5%)
TIL-rich IBC-NST	4 (2.0%)
Mucinous	5 (2.5%)
Lobular	15 (7.5%)
Micropapillary	3 (1.5%)
**Grade (*n*** **= 173)**	
I	3 (1.7%)
II	136 (78.6%)
III	34 (19.7%)
**Multicentricity**	
Positive	6 (3.0%)
Negative	194 (97.0%)
**Tumour size**	
T1	13 (6.5%)
T2	134 (67.0%)
T3	51 (25.5%)
T4	2 (1.0%)
**Lymph node**	
No	47 (23.5%)
N1	60 (30.0%)
N2	49 (24.5%)
N3	44 (22.0%)
**Tumour stage**	
I	4 (2.0%)
II	90 (45.0%)
III	106 (53.0%)
**Recurrence**	
Yes	61 (30.5%)
No	139 (69.5%)
**Time of follow up** **Median (Min-Max)**	50 (1–85)
**Time of recurrence** Median (Min-Max**)**	32 (1–85)
**RRM2 score**	
Negative	45 (22.5%)
Mild	10 (5.0%)
Moderate	86 (43.0%)
Intense	59 (29.5%)

The most common grade among invasive duct carcinoma cases was grade 2. The most common tumour stage was stage 3.

### RRM2 protein expression

**Fig. 1:**
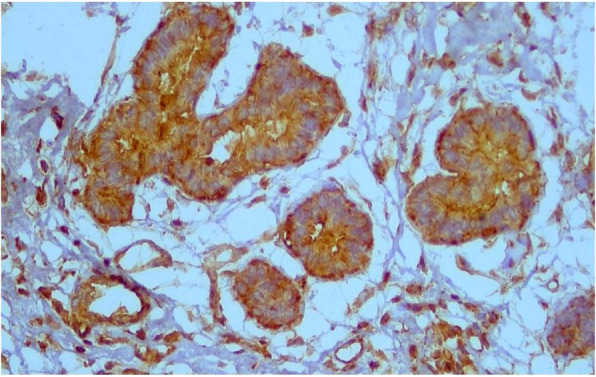
Intense RRM2 expression in cancer breast ×200

**Fig. 2:**
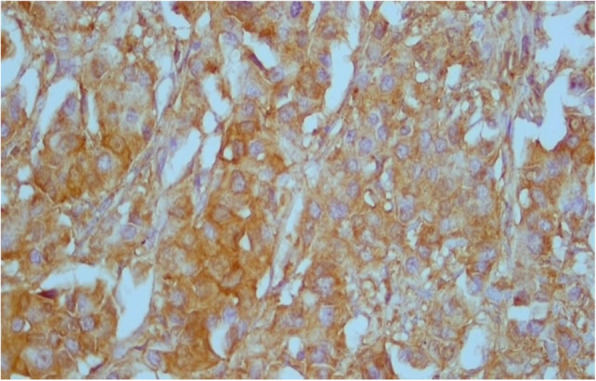
Moderate RRM2 expression in cancer breast × 400

**Fig. 3:**
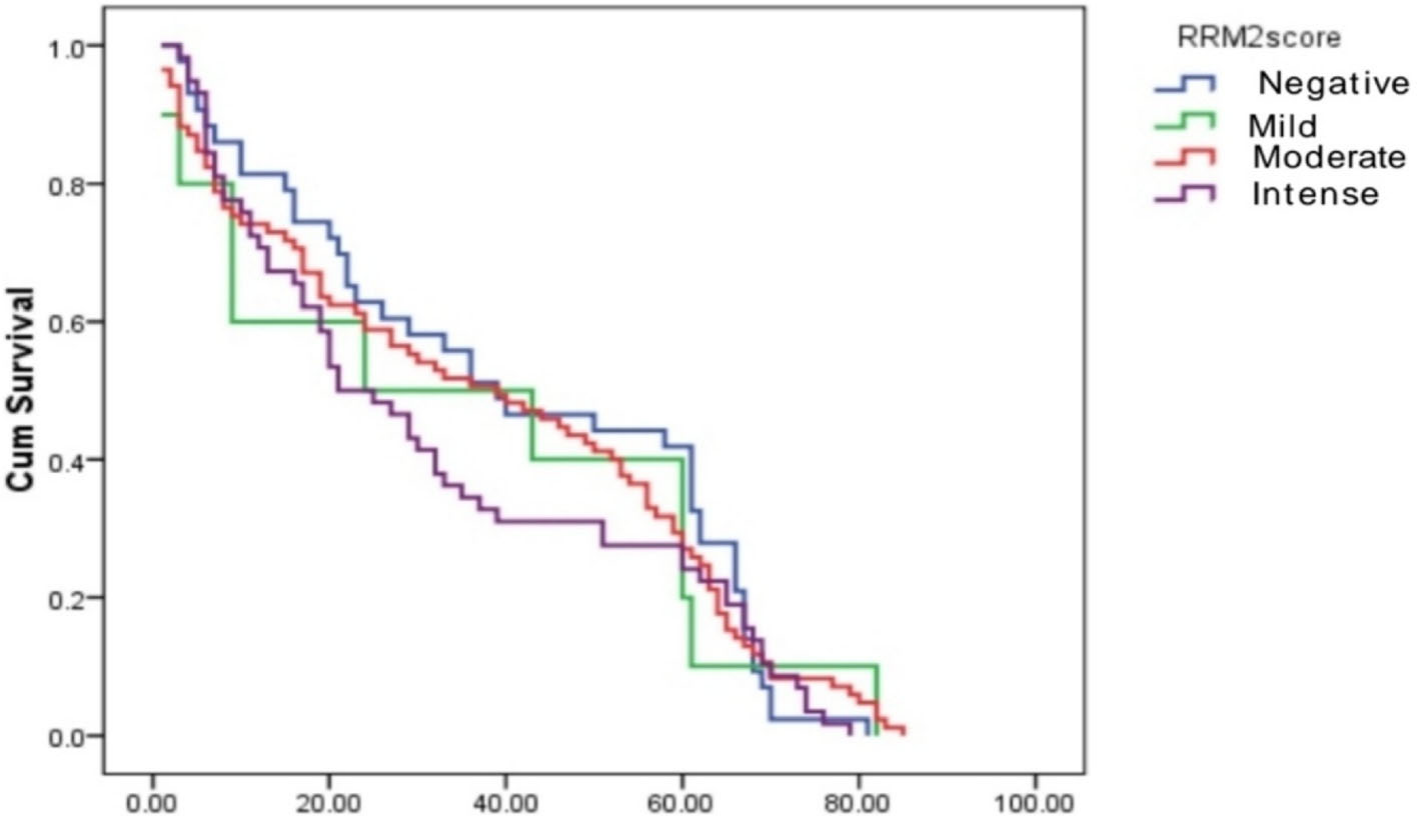
A Kaplan–Meier plot of RRM2 expression and DFS in whole series

**Fig. 4:**
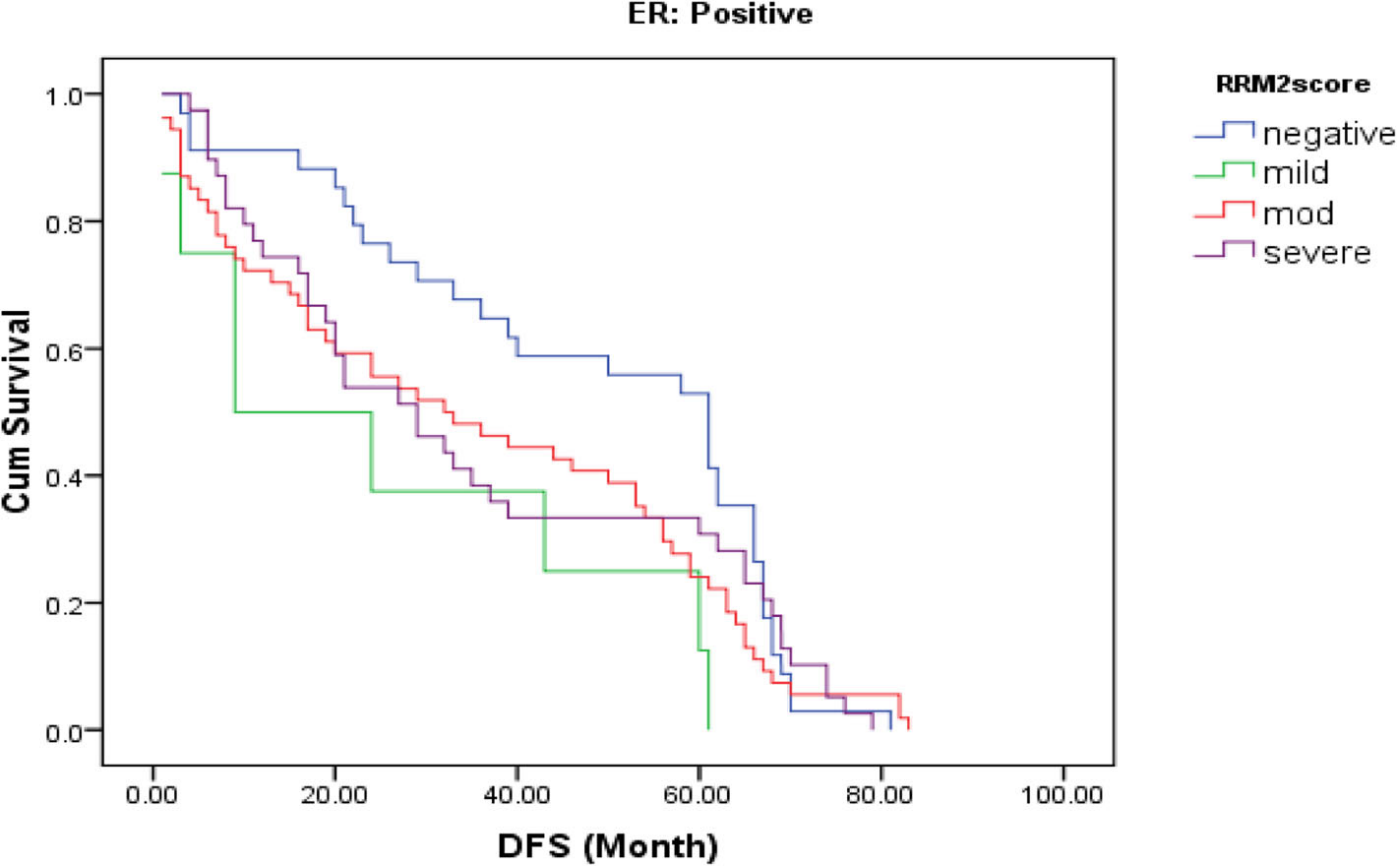
A Kaplan–Meier plot of RRM2 expression and DFS in ER-positive series

About 77% of cases were RRM2 positive (Fig 1 and 2). Correlation between RRM2 protein expression and the other clinicopathological variables are shown in (Table 2). In summary, moderate RRM2 score was common in both age groups (< 60 years and > 60 years). When we correlated RRM2 expression with ER, PR and HER2/neu expression, 85.5% of ER negative cases and 81.6% of PR negative cases showed RRM2 expression, while 73.9% of ER positive cases and 75% of PR positive cases showed RRM2 expression, this was not statistically significant. (84.4%) of HER2/neu positive cases expressed RRM2, while (75.8%) of HER2/neu negative cases expressed RRM2. High Ki67 median was observed in cases with high RRM2 score with statistical significance *(p* = 0.027). Among molecular subtypes of breast cancer, moderate RRM2 score was the most common score. Among different histological types of breast cancer, moderate RRM2 score was the most common score except in mucinous carcinoma where negative RRM2 score was the common score. Among cases of ductal carcinoma, moderate RRM2 score was common in high grade cases. Moderate RRM2 score was most common among all tumour stages. Intense RRM2 score was common in cases with recurrence (55.7%), and this was statistically significant (*p* < 0.001). Positive RRM2 expression group showed shorter disease free survival (Fig. 3 and Table 3). When we studied RRM2 expression in ER-positive group, we found that high RRM2 expression was associated with shorter disease free survival with borderline significance (*p* = 0.138) (Fig. 4 and Table 4).

**Table 2:** Correlation between RRM2 protein expression and other clinicopathological variables

	Total	RRM2 score				x^2^ (***p*** value)
		Negative	Mild	Moderate	Intense	
**Age/ years**						χ2 = 4.07 *P* = 0.254
< 60 y	109	21 (19.3%)	7 (6.4%)	44 (40.4%)	37 (33.9%)	
≥ 60 y	91	24 (26.4%)	3 (3.3%)	42 (46.2%)	22 (24.2%)	
**ER**						χ2 = 4.7 *P* = 0.196
Positive	138	36 (26.1%)	8 (5.8%)	54 (39.1%)	40 (29%)	
Negative	62	9 (14.5%)	2 (3.2%)	32 (51.6%)	19 (30.6%)	
**PR**						χ2 = 4.39 *P* = 0.22
Positive	124	31 (25.0%)	8 (6.5%)	47 (37.9%)	38 (30.6%)	
Negative	76	14 (18.4%)	2 (2.6%)	39 (51.3%)	21 (27.6%)	
**HER2/neu**						χ2 = 3.75 *P* = 0.312
Positive	45	7 (15.6%)	2 (4.4%)	18 (40.0%)	18 (40.0%)	
Negative	155	38 (24.2%)	8 (5.2%)	68 (43.9%)	41 (26.5%)	
**Ki67 median expression**	200	5 (2–40)	5 (5–40)	5 (5–50)	10 (2–60)	KW = 9.18 P = 0.027*
**Type**						MC *P* = 0.736
Luminal A	75	21 (28.0%)	3 (4.0%)	29 (38.7%)	22 (29.3%)	
Luminal B	40	9 (22.5%)	3 (7.5%)	16 (40.0%)	12 (30.0%)	
TN BLBC	39	9 (23.1%)	2 (5.1%)	16 (41.0%)	12 (30.8%)	
Her2 Luminal	17	2 (11.8%)	2 (11.8%)	8 (47.1%)	5 (29.4%)	
Her2 enriched	29	4 (13.8%)	0 (0%)	17 (58.6%)	8 (27.6%)	
**EGFR**						χ2 = 4.62 *P* = 0.202
Positive	150	39 (26.0%)	8 (5.3%)	61 (40.7%)	42 (28.0%)	
Negative	50	6 (12.0%)	2 (4.0%)	25 (50.0%)	17 (34.0%)	
**Histological type**						MC *P* = 0.326
Ductal	173	39 (22.5%)	9 (5.2%)	76 (43.9%)	49 (28.3%)	
TIL-rich IBC-NST	4	0 (0%)	1 (25.0%)	2 (50.0%)	1 (25.0%)	
Mucinous	5	3 (60.0%)	0 (0%)	1 (20.0%)	1 (20.0%)	
Lobular	15	2 (13.3%)	0 (0%)	7 (46.7%)	6 (40.0%)	
Micropapillary	3	1 (33.3%)	0 (0%)	0 (0%)	2 (66.7%)	
**Grade (n = 173)**						χ2 = 0.757 *P* = 0.847
I & II	139	33 (23.7%)	10 (7.2%)	46 (33.1%)	50 (36.0%)	
III	34	7 (20.6%)	1 (2.9%)	17 (50.0%)	9 (26.5%)	
**Multicent**						MC *P* = 0.629
Positive	6	1 (16.7%)	1 (16.7%)	2 (33.3%)	2 (33.3%)	
Negative	194	44 (22.7%)	9 (4.6%)	84 (43.3%)	57 (29.4%)	
**Tumour size**						χ2 = 0.685 *P* = 0.877
T1& 2	147	34 (23.1%)	7 (4.8%)	61 (41.5%)	45 (30.6%)	
T3 & 4	53	11 (20.8%)	3 (5.7%)	25 (47.2%)	14 (26.4%)	
**Lymph node**						χ2 = 4.53 *P* = 0.209
No & N1	107	21 (19.6%)	8 (7.5%)	49 (45.8%)	29 (27.1%)	
N2 & N3	93	24 (25.8%)	2 (2.2%)	37 (39.8%)	30 (32.3%)	
**Tumour stage**						χ2 = 4.89 *P* = 0.180
I & II	94	21 (22.3%)	8 (8.5%)	40 (42.6%)	25 (26.6%)	
III	106	24 (22.6%)	2 (1.9%)	46 (43.4%)	34 (32.1%)	
**Recurrence**						χ2 = 29.7 P ≤ 0.001*
Yes	61	9 (14.8%)	3 (4.9%)	15 (24.6%)	34 (55.7%)	
No	139	36 (25.9%)	7 (5.0%)	71 (51.1%)	25 (18.0%)	

χ2: Chi square test, *KW* Kruskil Wallis test, *MC* Monte Carlo test, *significant *p* ≤ 0.05

**Table 3:** Kaplan-Meier disease free survival for RRM2 score in whole series

	DFS				
	Median Survival time	Std. Error	95% CI	Log Rank test	***p*** value
RRM2score				1.14	0.767
Negative	40.558	3.820	33.07–48		
Mild	35.200	9.340	16.89–53.5		
Moderate	37.706	2.888	32.04–43.4		
Intense	32.483	3.297	26.02–38.9		
Overall DFS	36.65	1.85

**Table 4:** Kaplan-Meier DFS for RRM2 score in ER positive group

			DFS		
	Median Survival time	Std. Error	95% CI	Log Rank test	***P***-value
**RRM2 score**				5.51	0.138
Negative	47.559	3.962	39.79–55.32		
Mild	26.250	8.870	8.86–43.63		
Moderate	35.537	3.566	28.54–42.52		
Intense	35.590	4.122	27.51–43.66		
DFS	38.030	2.210	33.69–42.36

## Discussion

Breast cancer is a common type of cancer worldwide [21]. RRM2 is one of the important prognostic markers in cancer, it was found to be associated with large tumour size, positive lymph nodes and shorter survival in breast cancer [1, 11]. In the current study, percentage of high RRM2 expression in patients below 60 years of age was 33.9% and this was statistically insignificant. This was in agreement with Zhang et al. [1] who reported that high expression of RRM2 is not associated with age. Moderate and high RRM2 expression were common in cases with large tumour size and high lymph node stage, in agreement with other studies [1].

In this study, high RRM2 scores was more common in both TNBLBC and HER2/neu enriched types, representing 71.8 and 86.2% of the cases respectively. This was in agreement with Zhang et al. [1]. About 85.5% of ER negative cases and 81.6% of PR negative cases showed RRM2 expression versus 73.9% of ER positive cases and 75% of PR positive cases which showed RRM2 expression. Although this was statistically insignificant, it was in agreement with Chen et al. [22] who found ER and PR were negatively correlated with RRM2 expression.

When we studied RRM2 expression in ER positive group, we found that RRM2 expression was associated with shorter disease free survival with borderline significance, this was in agreement with Putluri et al. [23]. This could be explained by the role of RRM2 in tamoxifen resistance [9]. In this context, Zhang el al [1] found that in ER-negative breast cancers, RRM2 showed more prognostic power in comparison to ER-positive breast cancers possibly explained by its potential effect on Tamoxifen resistance [8, 9].

In our study, about 84.4% of HER2/neu positive cases expressed RRM2, while 75.8% of HER2/neu negative cases expressed RRM2. This was in agreement with Chen et al. [22] who reported that HER2/neu is positively related to RRM2.

RRM2 expression was significantly correlated with expression of Ki67. This was in agreement with Aird et al. study [6] who found that knocking down of RRM2 decreased cell growth and proliferation in epithelial tumours. It is known that RRM2 has an important role during S phase of cell cycle during DNA replication and this issue can have a potential therapeutic significance. Hence, several inhibitors of RRM2 have been developed for treatment of several cancer types including breast cancer [24, 25].

In conclusion, expression RRM2 and its correlation with clinicopathological parameters could help in evaluating outcome in breast cancer especially in ER-positive subgroup and can be a potential therapeutic target in actively proliferating tumours.

## Data Availability

All data are included in the manuscript and available upon reasonable request.
